# Supplementary material for: Subwavelength dielectric waveguide for efficient travelling-wave magnetic resonance imaging
Source: Nat Commun. 2024 Mar 14;15:2298. doi: 10.1038/s41467-024-46638-5 (PMC10940709; doi:10.1038/s41467-024-46638-5)
Supplement: Supplementary file 1 — Supplementary Information [file 41467_2024_46638_MOESM1_ESM.pdf]

Supplementary Information

Subwavelength Dielectric Waveguide for Efficient Travelling-Wave Magnetic Resonance Imaging

Yang Gao[1,2,3*], Tong Liu[1], Tao Hong[1,2], Youtong Fang[3], Wen Jiang[1,2], Xiaotong Zhang[3,4,5,6*]

[1]Hangzhou Institute of Technology, Xidian University, Hangzhou, China
[2]School of Electronic Engineering, National Key Laboratory of Antennas and Microwave Technology, Xidian University, Xi'an, China
[3]College of Electrical Engineering, Zhejiang University, Hangzhou, China
[4]Second Affiliated Hospital of Zhejiang University School of Medicine, Hangzhou, China
[5]MOE Frontier Science Center for Brain Science and Brain-machine Integration, Zhejiang University, Hangzhou, China
[6]Interdisciplinary Institute of Neuroscience and Technology, School of Medicine, Zhejiang University, Hangzhou, China

*Corresponding Authors:
Xiaotong Zhang, Associate Professor, College of Electrical Engineering, Zhejiang University, Hangzhou, China (email: zhangxiaotong@zju.edu.cn)
Yang Gao, Assistant Professor, Hangzhou Institute of Technology, Xidian University, Hangzhou, China (email: gaoyang01@xidian.edu.cn)

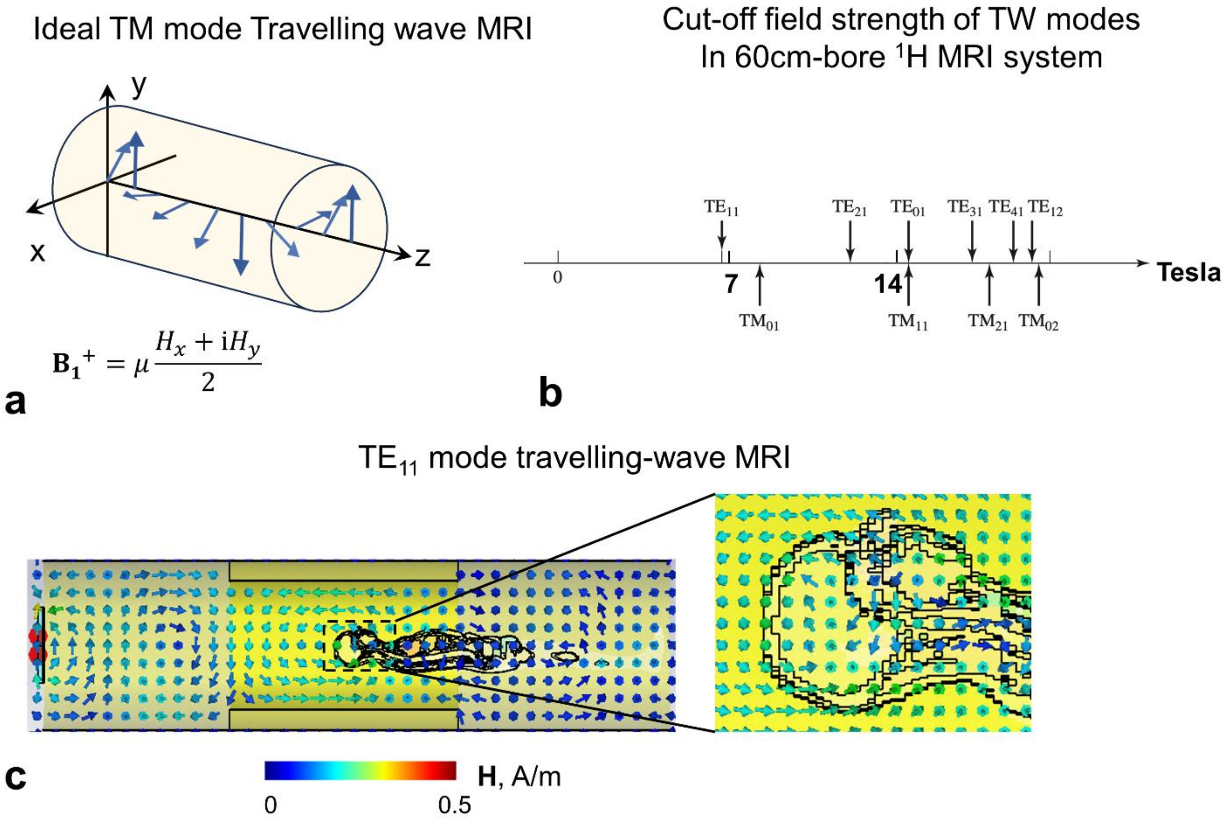

Supplement Fig.1 Canonical $\mathbf{TE_{11}}$ mode TW MRI. Only transverse magnetic field ($\mathbf{B_1^+}$) contributes to nuclei spin excitation (**a**). Cut-off limit for TM mode TW MRI (**b**). The vector plot of TE_{11} mode magnetic field. The primary mode TE11 is not optimal in nuclei spin excitation due to its prominent longitudinal magnetic field component (**c**).

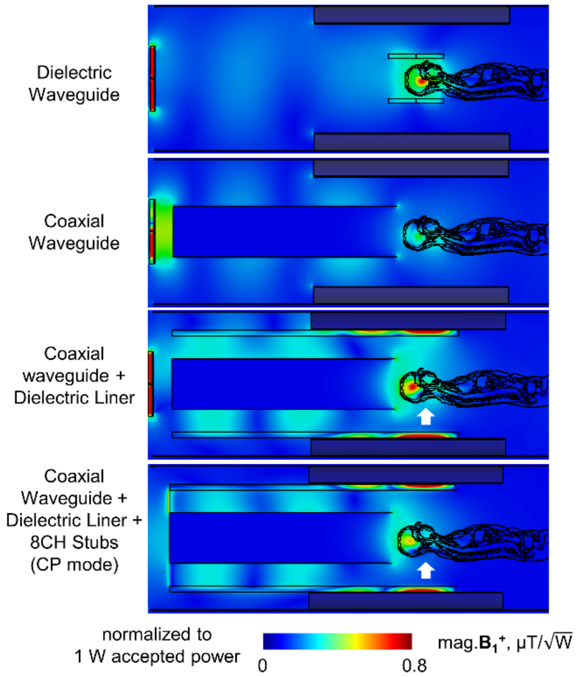

Transmission methods	MEAN. {mag.$\mathbf{B_1^+}$} (uT/$\sqrt{W}$)	$\sigma_{B_1^+}$ (uT/$\sqrt{W}$)	Max. SAR_{10g} (W/kg)	MEAN.{mag.$\mathbf{B_1^+}$}/ $\sqrt{\text{max. } SAR_{10g}}$ (uT/$\sqrt{W/kg}$)
Classic-TW	0.166	0.4199	0.125	0.469
Dielectric-Waveguide	0.3545	0.3777	0.331	0.616
Coaxial Waveguide	0.169	0.4124	0.1383	0.4544
Coaxial waveguide + Dielectric Liner	0.377	0.4881	0.431	0.5743
Coaxial Waveguide + Dielectric Liner + 8CH Stubs (CP mode)	0.294	0.4932	0.2636	0.5726

Supplement Fig. 2 Modified TW transmission vs. state-of-the-art implementations of TW transmission using single-channel mode. The distribution of $\mathbf{B_1^+}$ magnitude within the human subject **(left).** The statistics of performance in $\mathbf{B_1^+}$ efficiency, homogeneity, SAR as well as SAR normalized efficiency **(right).**
